# Clinical and ultrasound variables for early diagnosis of septic acute kidney injury in bitches with pyometra

**DOI:** 10.1038/s41598-020-65902-4

**Published:** 2020-06-02

**Authors:** Beatriz Gasser, Ricardo Andres Ramirez Uscategui, Marjury Cristina Maronezi, Letícia Pavan, Ana Paula Rodrigues Simões, Fernanda Martinato, Priscila Silva, Leandro Zuccolotto Crivellenti, Marcus Antônio Rossi Feliciano

**Affiliations:** 1 School of Agrarian Sciences and Veterinary Medicine, São Paulo State University “Julio de Mesquita Filho”, Jaboticabal, São Paulo, Brazil; 2 Institute of Agrarian Sciences, Federal University of the Jequitinhonha and Mucuri Valleys (UFVJM), Unaí, Minas Gerais, Brazil; 3 University of Franca (UNIFRAN), Franca, São Paulo, Brazil; 4 Federal University of Santa Maria, Santa Maria, Rio Grande do Sul, Brazil

**Keywords:** Nephrology, Kidney diseases, Inflammation, Acute inflammation, Sepsis

## Abstract

The aetiology of septic acute kidney injury (AKI) is not completely elucidated. Early identification of AKI in septic patients is considered to improve survival rate since it allows rapid treatment onset. We evaluated clinical, haematological, urinary, B-mode, spectral Doppler, and contrast-enhanced ultrasound variables in 20 bitches with pyometra as sepsis models and 12 healthy controls. All animals with pyometra presented some degree of renal damage on histological examination; however, sequential organ failure assessment (SOFA) classified only 40% cases with sepsis. AKI derived from systemic infection was identified in 57% of cases with hypoperfusion and in 22% with inflammation, being an affection of multifactorial origin. Among the evaluated parameters, urinary protein/creatinine ratio >0.15, serum albumin <2.94 mg/dL, time-averaged minimum velocity <21.5 cm/s, renal length/aorta diameter ratio >5.93, pulsatility index >1.53, haematocrit <45%, time-averaged maximum velocity <45.7 cm/s, haemoglobin <16 g/dL, leukocytes >12.53 × 10^3^/uL, and cortical contrast peak intensity <69%, in the order of accuracy, are significant indicators of septic AKI with an accuracy >80%. Thus, AKI is a very prevalent condition in septic patients, derived mainly from changes in renal perfusion and inflammation. Additionally, reviewing the SOFA score parameters is suggested to identify renal failure.

## Introduction

Sepsis is a life-threatening organ dysfunction caused by a dysregulated host response to infection, which causes high morbidity and mortality in humans and animals^1^, mainly due to poor tissue perfusion. The development of multiorgan dysfunction syndrome (MODS) affects approximately 50% of dogs with sepsis and increases the mortality rate by up to 70%^2^. Among the bacterial disorders affecting the canine species, pyometra is very common and results in sepsis development in 60% of confirmed cases^3^. Sepsis leads to acute kidney injury (AKI) in approximately 50% of people affected^4,5^ and in 12% of dogs, of which less than 14% survive^2,6^.

Pyometra is a disease caused by bacterial infection of the uterus, *E. coli* being the most common etiological agent, isolated in 90% of cases^7^. On average, 23 to 24% of bitches under 10 years old develop this condition^8^ and 50% above this age^9^. This disease can affect the kidneys at glomerular or tubular level, producing an acute or subacute lesion due to exacerbated stimulation of the immune system by the bacterial agent, which induces the formation of circulating immune complexes that precipitate in the glomeruli^10^.

The aetiology of AKI in patients with sepsis is complex, multifactorial, and unclear and is associated with changes in renal blood perfusion^4^, endothelial dysfunction, infiltration of inflammatory cells in the renal parenchyma, glomerular thrombosis, tubular obstruction by necrotic cells, or cellular debris^11^.

Although there is strong evidence to infer that AKI in patients with sepsis is due to changes in renal blood perfusion^4^, most studies in animals as experimental models only measured blood flow in the renal artery^11^, resulting in discrepant findings. For this reason, it is considered that this parameter does not explain reliably how the injury occurs, probably due to the complex nature of the renal vascularization^5^, or due to a significant proportion of other causal agents mentioned above. Therefore, considering the hemodynamic factor as the main pathological pathway to the lesion, recent experimental evidence using techniques that evaluate renal perfusion and not only blood flow allowed to infer that this phenomenon is caused by intrinsic alterations of the renal circulation^5,12^.

Early AKI identification in septic patients is considered to improve the survival rate since it allows rapid treatment onset. However, accurate tools for the early detection of this lesion are extremely invasive and puts the patient at high risk, such as renal biopsy, or are not available for routine clinical application such as scintigraphy, magnetic resonance imaging, and some new biochemical techniques^6^.

It is in this context that contrast-enhanced ultrasonography (CEUS) is presented as a promising diagnostic method. Wei and colleagues and Waller and colleagues opened the door for physiological and pathological studies using this technique for the renal perfusion estimation in dogs^13,14^. Schneider and colleagues demonstrated the accuracy of this method in humans to identify changes in renal perfusion induced by angiotensin^15^. Dong and colleagues, in a model of induced renal ischaemia in dogs, identified renal injury with CEUS, 30 days before abnormalities were detectable in routinely used blood biochemical parameters^16^. Finally, Lima and colleagues, in an experimental model of induced sepsis in pigs, identified CEUS changes in renal perfusion before and after treatment as accurately as with sublingual fluoroscopy^17^.

With these precepts, this clinical experimental study evaluated the clinical, haematological, urinary, ultrasonographic B-mode, spectral Doppler, and CEUS variables in 20 bitches with spontaneous pyometra, as experimental models of sepsis, and 12 healthy control patients, aiming to (1) identify the pathophysiological changes that may help understand sepsis-related AKI development, (2) verify the accuracy of these methods for AKI diagnosis using histopathological examination of renal biopsy as gold standard, and 3) evaluate the sequential organ failure assessment (SOFA) score parameters as identifiers of AKI in dogs with sepsis.

## Results

According to histopathological examination, 20/20 animals (100%) with pyometra presented some degree of tissue injury and more than one concomitant alteration, 20/20 (100%) tubular degeneration (TUBdeg), 14/20 (70%) inflammatory infiltrate (INFInf), 13/20 (65%) acute tubular necrosis (ATN), 13/20 (65%) interstitial fibrosis (INTFib), 9/20 (45%) membranoproliferative glomerulonephritis (MPGN), and 4/20 (20%) membranous glomerulonephritis (MGN). According to these results, all animals with pyometra were diagnosed with AKI.

On physical examination, only the heart rate (HR) was higher (p < 0.01) in patients with AKI. Regarding haematological parameters, haematocrit, haemoglobin, erythrocytes, and albumin levels were lower (p < 0.01) in animals with AKI, whereas leukocytes, globulin, and alkaline phosphatase (ALP) levels were higher (p < 0.01). Upon urinary examination, the urinary protein/creatinine ratio (UP/CR) was higher (p < 0.01) in patients with AKI. Upon haemogasometry analysis, arterial bicarbonate concentration (CaHCO_3_) was lower (p < 0.01) (Table 1).

**Table 1:** Mean ± SD and statistical results (t-Student and least squares regression) of physiological, haematological, and urinary parameters in healthy and acute kidney injury secondary to sepsis/pyometra bitches.

Parameter	Healthy patients	AKI patients	P-value	Regression coefficient	Probability ratio
**Physical exam**					
HR (bpm)	108 ± 26.3	131 ± 39.5	0.0025*	0.28	1.32
*f*_R_ (rpm)	21.5 ± 24.0	36.0 ± 31.0	0.0201	N/A	N/A
GCS	18.0 ± 0.00	18.0 ± 1.00	0.0396	N/A	N/A
Temperature (°C)	38.5 ± 0.77	38.8 ± 0.77	0.6253	N/A	N/A
SAP (mmHg)	115.8 ± 23.8	115 ± 32.5	0.8607	N/A	N/A
**Hemogram**					
Haematocrit (%)	48.9 ± 3.50	41.3 ± 15.1	0.0005*	−2.96	0.07
Haemoglobin (g/dL)	17.1 ± 1.25	14.2 ± 5.40	0.0019*	−2.31	0.10
Erythrocytes (x10^6^/uL)	6.96 ± 0.51	5.87 ± 2.17	0.0060*	−0.06	1.00
Leukocytes (x10^3^/uL)	10.2 ± 3.39	20.8 ± 27.7	0.0090*	0.47	1.23
Neutrophils (x10^3^/uL)	7.70 ± 3.07	15.9 ± 20.6	0.0239	N/A	N/A
Monocytes (x10^3^/uL)	0.31 ± 0.28	0.79 ± 1.90	0.0239	N/A	N/A
Band cells (x10^3^/uL)	0.00 ± 0.02	0.07 ± 0.73	0.0399	N/A	N/A
Lymphocytes (x10^3^/uL)	1.78 ± 0.95	2.39 ± 0.25	0.1498	N/A	N/A
Eosinophils (x10^3^/uL)	0.38 ± 0.20	0.19 ± 0.50	0.2252	N/A	N/A
MCV (fL)	70.1 ± 0.67	67.9 ± 5.72	0.2429	N/A	N/A
MCH (pg)	24.5 ± 0.25	24.4 ± 1.73	0.4636	N/A	N/A
MCHC (g/dL)	34.9 ± 0.12	35.1 ± 1.66	0.6404	N/A	N/A
Platelets (x10^3^/uL)	323 ± 77.7	322 ± 203	0.8763	N/A	N/A
Basophils (x10^3^/uL)	0.00 ± 0.00	0.00 ± 0.00	1.0000	N/A	N/A
**Biochemical**					
Albumin (g/dL)	3.46 ± 0.37	0.39 ± 0.82	0.0002*	−3.57	0.03
Globulins (g/dL)	3.92 ± 1.30	5.80 ± 1.59	0.0004*	0.49	1.63
ALP (U/L)	53.0 ± 22.6	184 ± 144	0.0053*	0.23	1.00
ALT (U/L)	43.0 ± 8.67	23.00 ± 25.50	0.0233	N/A	N/A
Creatinine (mg/dL)	1.20 ± 0.10	1.00 ± 0.54	0.0423	N/A	N/A
Total Protein (g/dL)	7.58 ± 0.97	8.20 ± 1.52	0.1191	N/A	N/A
TBilirubin (mg/dL)	0.32 ± 0.23	0.20 ± 0.15	0.1265	N/A	N/A
UBilirubin (mg/dL)	0.06 ± 0.08	0.09 ± 0.07	0.1279	N/A	N/A
CBilirubin (mg/dL)	0.08 ± 0.03	0.09 ± 0.09	0.7240	N/A	N/A
Glycaemia (mg/dL)	88.5 ± 17.8	92.5 ± 21.8	0.5202	N/A	N/A
Urinalysis					
UP/CR	0.06 ± 0.07	0.39 ± 0.82	0.0001*	9.80	3.68
Density	1.033 ± 0.01	1.020 ± 0.01	0.1720	N/A	N/A
**Hemogasometry**					
CaHCO_3_ (mmol/L)	19.2 ± 1.37	18.1 ± 2.72	0.0064*	−0.19	0.88
PaO_2_ (mmHg)	97.2 ± 7.58	69.7 ± 53.2	0.0323	N/A	N/A
PaO_2_/FiO_2_	462 ± 36.1	331 ± 253	0.0323	N/A	N/A
PaCO_2_ (mmHg)	33.4 ± 3.97	29.9 ± 8.00	0.0429	N/A	N/A
pH	7.39 ± 0.03	7.39 ± 0.05	0.7702	N/A	N/A

SD: standard deviation, AKI: Acute kidney injury, HR: Heart rate, bpm: beats per minute; *f*_R_: Respiratory rate, rpm: Respirations per minute; GCS: Glasgow coma scale, SAP: Systolic arterial pressure, MCV: Mean corpuscular volume, MCH: mean corpuscular haemoglobin, MCHC: mean corpuscular haemoglobin concentration, ALP: alkaline phosphatase, ALT: Alanine aminotransferase, TBilirubin: Total bilirubin, UBilirubin: Unconjugated bilirubin, CBilirubin: Conjugated bilirubin, UP/CR: urinary protein/creatinine ratio, CaHCO_3_: arterial bicarbonate concentration, PaO_2_: arterial partial pressure of oxygen, FiO_2_: Fraction inspired of oxygen, PaCO_2_ arterial partial pressure of carbon dioxide, N/A: Not applicable. *Considered statistically significant by t-Student test, (P ≤ 0.01).

In the B-mode ultrasonographic evaluation (Table 2), the renal length/aorta diameter ratio (RLeng/AorRatio) was higher (p < 0.01) in patients with AKI. At Doppler flowmeter, renal blood flow (RBF), time-averaged minimum velocity (TaMin), and time-averaged maximum velocity (TaMax) were lower in patients with AKI, while pulsatility and resistivity indexes were higher (p < 0.01). Among the parameters evaluated by CEUS, only cortical peak contrast enhancement (CortPeak) was lower (p = 0.01) in patients with AKI.

**Table 2:** Mean ± SD of the parameters evaluated in the left kidney by different ultrasonographic methods (B-Mode, Doppler and contrast enhancement ultrasound CEUS) in healthy and affected by acute kidney injury secondary to sepsis/pyometra bitches.

Parameter	Healthy patients	AKI patients	P-value	Regression coefficient	Probability ratio
**B-Mode**					
RLeng/AorRatio	5.55 ± 0.43	6.90 ± 1.68	0.0012*	8.45	4.75
Cort/MedRatio	1.07 ± 0.21	0.99 ± 0.20	0.0797	N/A	N/A
**Doppler Ultrasonography**					
Pulsatility index	1.23 ± 0.38	1.81 ± 1.54	0.0004*	2.96	1.98
TaMin (cm/s)	26.17 ± 9.47	17.8 ± 10.83	0.0005*	−4.26	0.01
TaMax (cm/s)	52.65 ± 20.12	34.52 ± 21.08	0.0005*	−2.43	0.09
Resistivity index	0.66 ± 0.10	0.74 ± 0.12	0.0057*	1.19	1.60
RBF (mL/min/g)	1.61 ± 0.54	1.02 ± 0.94	0.0100*	−1.67	0.27
SystVel (cm/s)	99.55 ± 33.65	82.15 ± 30.27	0.0102	N/A	N/A
DiastVel (cm/s)	36.73 ± 11.50	22.42 ± 16.41	0.0396	N/A	N/A
**CEUS**					
CortPeak (%)	96.51 ± 58.02	37.20 ± 84.70	0.0098*	−1.81	0.02
MedPeak (%)	122.40 ± 48.10	75.30 ± 73.20	0.0360	N/A	N/A
Cort(a) (%/s)	1.43 ± 2.02	2.91 ± 1.65	0.0429	N/A	N/A
MedAUC	1119 ± 706	1503 ± 1448	0.1857	N/A	N/A
Med(a) (%/s)	0.72 ± 0.80	0.87 ± 0.68	0.2758	N/A	N/A
CortTP (s)	15.71 ± 7.92	11.67 ± 4.01	0.2933	N/A	N/A
CortAUC	518.00 ± 449.00	836.00 ± 1035.00	0.3305	N/A	N/A
Med(b) (%/s)	0.58 ± 0.62	0.64 ± 0.42	0.4596	N/A	N/A
Cort(b) (%/s)	0.98 ± 0.99	1.46 ± 0.92	0.5334	N/A	N/A
MedTP (s)	41.86 ± 21.23	39.46 ± 21.79	0.5334	N/A	N/A
CortMtT (s)	20.70 ± 13.93	22.41 ± 12.73	0.6404	N/A	N/A
MedMtT (s)	51.20 ± 23.70	49.36 ± 26.57	0.8457	N/A	N/A

SD: standard deviation, AKI: Acute kidney injury, RLeng/AorRatio: Renal length/aorta diameter ratio, Cort/MedRatio: Corticomedullary ratio, TaMin: time-averaged minimum velocity, TaMax: time-averaged maximum velocity, RBF: Renal blood flow, SystVel: Systolic velocity, DiastVel: Diastolic velocity, Cort: Cortical, MED: Medullary, Peak: peak contrast enhancement, TP: time to peak, MtT: mean transmission time, AUC: area under the curve, (a): slope of the input curve and (b): slope of output curve, N/A: Not applicable. *Considered statistically significant by t-Student test, (P ≤ 0.01).

**Figure 1:**
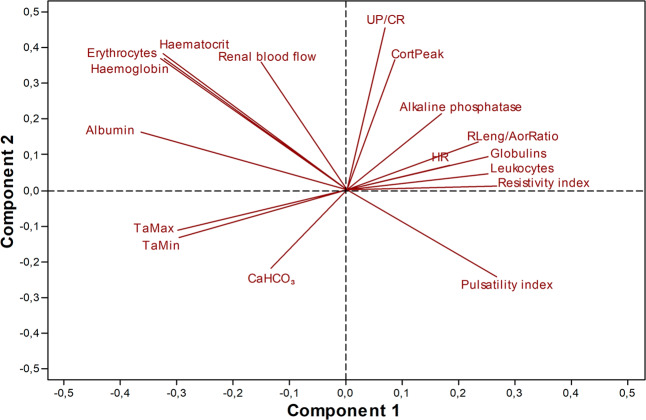
Loading plot of the partial least squares’ discriminant analysis model of regression, for acute kidney injury secondary to sepsis/pyometra in bitches and clinical predictors studied.

When assessing the relationship between AKI and the various predictors studied (17 variables with significant results between healthy patients and those with AKI), by the least squares regression, it determined that the association of these variables allows to create a model with a coefficient of determination of 82% (Fig. 1), but only the variables UP/CR, albumin, TaMin, RLeng/AorRatio, pulsatility index, haematocrit, TaMax, haemoglobin, leukocytes, and CortPeak were considered significant predictors (p < 0.01). Their regression coefficients and probability ratios are described in Tables 1 and 2.

**Figure 2:**
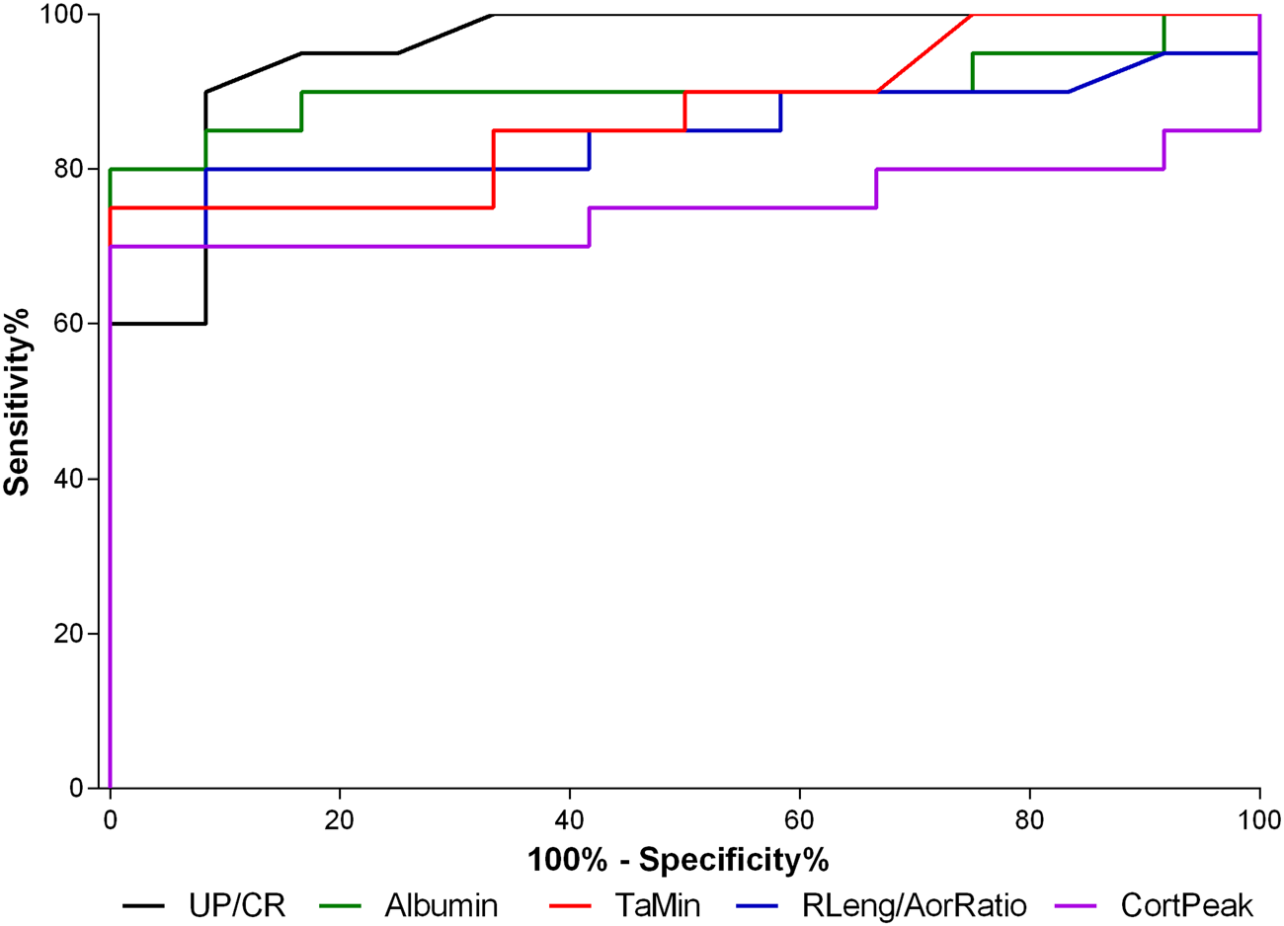
Receiving operating characteristic (ROC) curves comparing the diagnostic sensitivity and specificity of the different laboratory and ultrasound parameters studied (accuracy > 80%) for acute kidney injury secondary to sepsis/piometra identification in bitches.

The results of discriminative power analysis for AKI identification in bitches affected by pyometra/sepsis are shown in Table 3. Further, the comparative study of receiver operating characteristic curves (ROC) is shown in Fig. 2. Among these parameters, UP/CR, albumin, TaMin, RLeng/AorRatio, pulsatility index, haematocrit, TaMax, haemoglobin, leukocytes, and CortPeak are considered to have significant diagnostic value (p < 0.05 and accuracy >80%).

**Table 3:** Diagnostic performance variables of different ultrasound, clinical and laboratory parameters to predict acute kidney injury in bitches affected by sepsis/pyometra.

Parameter	Cutoff point	Sensibility%	Specificity%	Accuracy%	Likelihood ratio	DiagAUC%
**B-Mode**						
RLeng/AorRatio	>5.93	80	91.67	84.38	9.6	84.79
**Doppler Ultrasonography**						
Pulsatility index	>1.53	70	91.67	81.25	8.4	87.90
TaMin (cm/s)	<21.50	75	91.67	84.38	9.0	87.08
TaMax (cm/s)	<45.70	80	83.30	81.25	4.8	87.80
Resistivity index	>0.71	70	83.30	75.00	4.2	80.42
RBF (ml/min/g rim)	<1.45	70	66.70	68.75	2.1	76.30
**CEUS**						
CortPeak (%)	<69.00	70	98.00	81.25	8.4	75.00
**Physical Exam**						
HR (bpm)	>123	70	75.00	75.00	2.8	82.30
**Hemogram**						
Haematocrit (%)	<45	75	91.67	81.25	9.0	87.08
Haemoglobin (g/dL)	<16	73	83.33	81.25	4.5	83.13
Erythrocytes (x10^6^/uL)	<6.64	70	76.00	75.00	2.8	79.83
Leukocytes (x10^3^/uL)	>12.53	75	91.67	81.25	9.0	77.50
**Biochemical**						
Albumin (g/dL)	<2.94	85	91.67	87.50	10.2	90.42
Globulins (g/dL)	>4.90	75	83.30	81.25	4.5	87.71
**Urinalysis**						
UP/CR	>0.15	90	91.67	90.63	10.8	95.42
**Haemogasometry**						
CaHCO_3_ (mmol/L)	<18.6	80	83.30	81.25	4.8	79.20

UP/CR: urinary protein/creatinine ratio, CaHCO_3_: arterial bicarbonate concentration, RLeng/AorRatio: Renal length/aorta diameter ratio, TaMin: time-averaged minimum velocity, TaMax: time-averaged maximum velocity, RBF: Renal blood flow, CortPeak: Cortical peak contrast enhancement, DiagAUC: Area under the curve of receiving operating characteristic.

**Figure 3:**
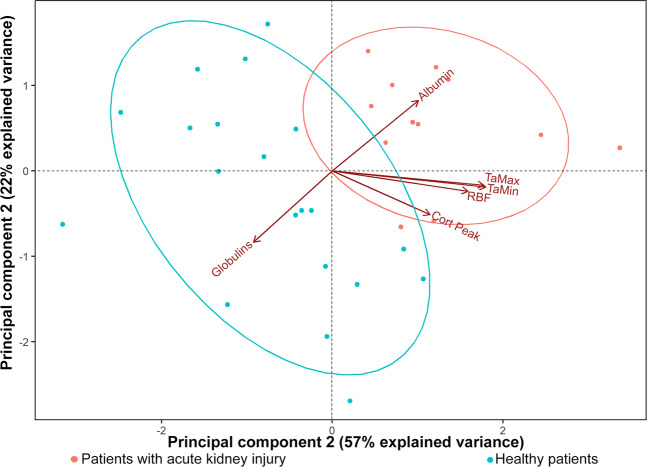
Graphical representation of principal components analysis involved in the development of acute kidney injury secondary to sepsis/pyometra in bitches.

The principal component analysis (PCA) included all those parameters that were significant when comparing the animals with AKI and without AKI; subsequently, the parameters were filtered until they were able to fit at least 70% of the variability into two components that explain AKI in this clinical trial. In this way, it was possible to obtain two main components that explain 79% of the changes resulting in AKI. Component 1 (Fig. 3 X-axis) corresponding to 57% of the cases, represented by the negative alteration of the RBF, TaMax, TaMin, and CortPeak in patients with AKI, infers that it is the hemodynamic component of the lesion or the result of tissue hypoperfusion. The second component, associated with albumin reduction and globulins increase (Fig. 3 Y-axis), represents 22% of cases with AKI and is related to inflammation.

All parameters studied were correlated with the degrees of renal lesion found on histopathological examination (considering p < 0.05 and Spearman’s rho < −0.5 or >0.5; Table 4). As a result of this analysis, albumin reduction corresponds to worsening degree of TUBdeg, INTFib, and INFInf; PaO_2_ reduction indicates worsening degree of MPGN; and reduction of TaMax, TaMin, and haematocrit is related to worsening degree of TUBdeg, while the increase in UP/CR is related to a higher degree of ATN, TUBdeg, INTFib, and INFInf, increase in the RLeng/AorRatio worsens ATN, and increase in ALP and pulsatility index indicates higher degrees of TUBdeg.

**Table 4:** Results of significant Spearman’s correlation analysis between clinical, laboratory and ultrasonographic parameters evaluated and the degrees of renal histopathological alterations in bitches with acute kidney injury secondary to sepsis/pyometra.

Parameter	Statistical results	Histopathologic renal lesions degree					
		ATN	TUBdeg	INTFib	INFInf	MGN	MPGN
Haematocrit (%)	P-value	0,0153	0,0008	0,0328	0,0076	0,8624	0,1339
	r-Spearman	−0,42	−0,56	−0,38	−0,46	−0,03	−0,27
Albumin (g/dL)	P-value	0,0059	0,0002	0,0029	0,0009	0,4402	0,0130
	r-Spearman	−0,48	−0,61	−0,51	−0,56	−0,14	−0,43
ALP (U/L)	P-value	0,1421	0,0009	0,0144	0,0175	0,2417	0,0219
	r-Spearman	0,27	0,56	0,43	0,42	0,21	0,40
PaO_2_ (mmHg)	P-value	0,0619	0,0107	0,5416	0,0732	0,4371	0,0033
	r-Spearman	−0,33	−0,44	−0,11	−0,32	−0,14	−0,51
UP/CR	P-value	0,0028	0,0000	0,0003	0,0000	0,0127	0,0055
	r-Spearman	0,51	0,78	0,60	0,66	0,44	0,48
RLeng/AorRatio	P-value	0,0030	0,0245	0,1927	0,4609	0,1052	0,9596
	r-Spearman	0,51	0,40	0,24	0,14	0,29	−0,01
TaMax (cm/s)	P-value	0,0560	0,0056	0,0778	0,0740	0,7364	0,5023
	r-Spearman	−0,34	−0,56	−0,32	−0,32	−0,06	−0,12
TaMin (cm/s)	P-value	0,0619	0,0049	0,0781	0,0334	0,7363	0,3167
	r-Spearman	−0,33	−0,60	−0,32	−0,38	−0,06	−0,18
Pulsatility index	P-value	0,1076	0,0011	0,1084	0,0495	0,9158	0,6434
	r-Spearman	0,29	0,55	0,29	0,35	0,02	0,09

ATN: acute tubular necrosis, TUBdeg: tubular degeneration, INTFib: interstitial fibrosis, INFInf: inflammatory infiltrate, MPGN: membranoproliferative glomerulonephritis, MGN: membranous glomerulonephritis, ALP: alkaline phosphatase, UP/CR: urinary protein/creatinine ratio, PaO_2_: arterial partial pressure of oxygen, RLeng/AorRatio: Renal length/aorta diameter ratio, TaMin: time-averaged minimum velocity, TaMax: time-averaged maximum velocity.

Of the 20 animals with pyometra, 8 (40%) had a SOFA score greater than 2 and, consequently, were diagnosed with sepsis. Based on the results of this score evaluation, dysfunction of the following systems was found: renal in 4/20 (20%) patients evaluated, respiratory 4/20 (20%), coagulation 3/20 (15%), cardiovascular 2/20 (10%), neurological 2/20 (10%), and hepatic 1/20 (10%). In addition, the resulting values of UP/CR, lymphocytes, neutrophils, band cells, pulsatility index, monocytes, and urea were higher (p < 0.05), while the values of the corticomedullary ratio (Cort/MedRatio), CaHCO_3_, haemoglobin, haematocrit, erythrocytes, ALT, and arterial pH were lower (p < 0.05) in patients with sepsis (Table 5). Moreover, it was possible to associate sepsis with more severe cases of interstitial fibrosis and membranoproliferative glomerulonephritis (p = 0.023 and 0.015, respectively) among the renal lesions found.

**Table 5:** Mean ± SD of physiological, haematological, urinary and ultrasound parameters significantly different (p < 0.01) in female dogs with and without sepsis secondary to pyometra.

Parameter	Sepsis	Non sepsis	P-value
**Hemogram**			
Lymphocytes (x10^3^/uL)	3.34 ± 1.94	1.49 ± 1.49	0.0060*
Neutrophils (x10^3^/uL)	28.15 ± 23.33	10.85 ± 9.98	0.0150*
Band cells (x10^3^/uL)	0.84 ± 1.95	0.00 ± 0.13	0.0230*
Haemoglobin (g/dL)	11.80 ± 6.63	15.65 ± 3.48	0.0320*
Haematocrit (%)	33.90 ± 18.95	45.45 ± 7.75	0.0350*
Monocytes (x10^3^/uL)	1.05 ± 2.37	0.42 ± 0.93	0.0460*
Erythrocytes (x10^6^/uL)	4.75 ± 3.16	6.58 ± 1.36	0.0550
**Biochemical**			
ALT (U/L)	20.00 ± 15.75	38.50 ± 28.80	0.0550
Urea (mg/dL)	72.50 ± 90.00	29.50 ± 19.25	0.0960
**Urinalysis**			
UP/CR	1.02 ± 0.80	0.19 ± 0.36	0.0150*
**Haemogasometry**			
CaHCO_3_ (mmol/L)	16.09 ± 5.48	18.59 ± 1.40	0.0200*
pH	6.15 ± 1.38	6.50 ± 1.00	0.0960
**Ultrasonography**			
Cort/MedRatio	0.87 ± 0.08	1.07 ± 0.30	0.0130*
Pulsatility index	2.58 ± 2.63	1.41 ± 1.00	0.0400*
UterineDiam (cm)	2.63 ± 3.16	1.60 ± 2.59	0.0900

ALT: Alanine aminotransferase, UP/CR: urinary protein/creatinine ratio, CaHCO_3_: arterial bicarbonate, Cort/MedRatio: Corticomedullary ratio, UterineDiam: Uterine diameter. *Considered statistically significant by Mann-Whitney test (P ≤ 0.05).

## Discussion

All patients with pyometra had some degree of AKI caused by different histopathological lesions, although only 40% of them had signs compatible with sepsis through SOFA score evaluation. Renal impairment in this study could be explained in 57% of cases by changes in renal hemodynamic, mainly due to reduced magnitude and velocity of RBF assessed by Doppler ultrasonography and cortical perfusion assessed by peak contrast enhancement in the CEUS evaluation. In another 22% of cases, the lesion was explained by systemic inflammatory disease, associated with a reduction in plasma albumin concentration and an increase in globulins. Previous studies have shown that endotoxemia leads to AKI due to renal microcirculation dysfunction caused by decreased perfusion, oxygen distribution, and adherence of cytokines and leukocytes to the vascular endothelium resulting from inflammation^18,19^.

Among the studied diagnostic variables, UP/CR, albumin, TaMin, RLeng/AorRatio, pulsatility index, TaMax, haematocrit, hemoglobin, leukocytes, and CortPeak exhibited an accurate predictive value for AKI diagnosis (>80% accuracy) in patients with sepsis/pyometra. Proteinuria has already been described as an early marker of renal injury and calculation of the UP/CR ratio has been identified as accurate in defining critical proteinuria values in humans^20^. Increased concentration of low-molecular-weight proteins in urine may be indicative of their excess within the proximal tubular cells or injury and dysfunction of these cells; these proteins should be completely reabsorbed by the proximal tubular cells^21^.

Adembri and colleagues demonstrated in an experimental model of sepsis in rats that during the initial stage of the condition, renal damage consisted of diffuse structural alterations of renal corpuscles and glomerular epithelium components, leading to increased albumin permeability and, consequently, elevation of urinary albumin/creatinine ratio up to three times the value found in healthy subjects, and through electrophoresis, it was shown that proteinuria was limited to albumin and low-molecular-weight proteins^22^. In contrast, proteinuria can also be observed in cases of bacterial lower urinary tract infection due to contamination of urine with bacterial proteins and leukocytes^23^; however, this study has no evidence that asymptomatic lower urinary tract infections may cause proteinuria or microalbuminuria.

Furthermore, the hypoalbuminemia observed in AKI patients can be attributed to both proteinuria caused by tubular cell injury and liver function alteration in the face of infection, leading hepatocytes to change their metabolic pathway in favour of the inflammatory response, causing an increase in acute-phase protein synthesis (globulins) and reduction in albumin synthesis^24^.

Although the present study did not determine which protein types were involved in proteinuria, the subtle increase in UP/CR values (>0.15) and the reduction in albumin (<2.94 g/dL) correlated significantly with the degree of tubular degeneration, acute tubular necrosis, inflammatory infiltrate and membranoproliferative glomerulonephritis (Table 4), corroborating with glomerular and tubular impairment. In addition, microscopic examination of urinary sediment in patients with pyometra rarely found bacteria and/or leukocytes, which precludes the possibility of sample contamination, reinforcing the accuracy of these laboratory analyses as early indicators of AKI, when compared with serum concentrations of urea and creatinine that are routinely used.

Dopplerfluxometric parameters are widely used in medicine as early indicators of kidney disease, in assessing the viability of kidney transplantation and their usefulness in dogs and cats^25–29^. In this context, resistive and pulsatility indexes are considered by the medical literature as the most accurate ultrasound parameters for AKI identification, differing from our results that indicate the average velocity times (TaMin and TaMax) as the most accurate. These measurements represent the minimum and maximum renal artery blood flow velocity in the Doppler spectrum in a cardiac cycle and, therefore, the average flow velocity in this vessel^30^. To our knowledge, these parameters have never been described as early indicators of AKI and our results identify that TaMin values <21.50 cm/s and TaMax <45.70 cm/s are indicative of AKI with an accuracy greater than 80%, and the reduction magnitude of these parameters is correlated with a higher degree of tubular degeneration.

Pulsatility and resistivity indexes indicate resistance to blood flow within the artery and their normal values have already been established for humans and animals, considering that, in non-sedated dogs, pulsatility index >1.52 and resistivity index >0.72 are indicative of abnormality^26^. In humans with septic shock, renal artery pulsatility index >1.55 and resistivity index >0.74 are indicative of severe renal dysfunction associated with rapid decline in renal function^25,31^. In the present study, the cutoff values of these indexes obtained for AKI determination were similar to those described in literature for humans and dogs (pulsatility index >1.53 and resistivity index >0.71) with high accuracy, indicating Doppler ultrasound as a valuable diagnostic tool for early sepsis-related AKI determination.

In B-mode ultrasonography, the assessment of the renal length/aorta diameter ratio was the parameter with the greatest accuracy for AKI determination. In dogs, the RLeng/AorRatio has been proven as a parameter to determine the renal size and is considered to be decreased when the ratio is <5.5 and increased when >9.1^32^. Kidney size increase can be seen in acute cases of proliferative glomerulonephritis, acute tubular necrosis and acute interstitial nephritis and is also expected in renal vein thrombosis, while kidney size decrease is observed in chronic kidney disease^33^. The present study showed a cutoff value >5.93 as indicative of AKI with accuracy above 80%, which mainly correlated with acute tubular necrosis, tubular degeneration and membranous glomerulonephritis.

In blood laboratory tests, the parameters haematocrit, haemoglobin, leukocytes, and CaHCO_3_ showed high accuracy in determining AKI. Reduced haematocrit and haemoglobin levels have been described as a risk factor for AKI aggravation in critically ill patients, associated with worsening renal tissue hypoxia^34^, in which activated leukocytes adhere to the renal endothelium, contributing to flow obstruction and release of proinflammatory cytokines, creating an inflammatory cycle in the microcirculation^19^. Accordingly, the reduction in the first two haematological parameters was associated with tubular degeneration, acute tubular necrosis and inflammatory infiltrate, and leukocytosis was also associated with worsening in these three types of lesions, besides membranous glomerulonephritis. Regarding bicarbonate, its utility in the diagnosis of AKI has already been described and subtle reduction is associated with worsening renal function^35^. Bicarbonate acts on tissue by increasing the availability of oxygen in the medullary region and reducing free radicals’ formation^36^. In metabolic acidosis, ammonia production occurs in the medullary region, leading to increased inflammation and tubular injury^37^. The present study demonstrated that serum bicarbonate value <18.6 mmol/L is related to AKI and worsening of the degree of tubular degeneration.

Among the CEUS parameters, only the decrease in cortical peak contrast enhancement <69% was significant for the diagnosis of AKI with >80% accuracy, associated with degeneration and acute tubular necrosis. Our data corroborate with Lima and colleagues, who found in pigs with septic shock a decrease in peak intensity that persisted even after volume resuscitation^17^. Legrand and colleagues proved that treatment of renal hypoperfusion in rats does not completely prevent microcirculatory dysfunction by endotoxemia and that it can occur even under normal macrovascular perfusion conditions^18^. These data reinforce the importance of renal microcirculation evaluation in critically ill patients by the CEUS technique, since normal macroperfusion values do not necessarily rule out renal parenchymal vascular impairment, although this was not observed in this clinical study.

Regarding animals considered to have sepsis by the SOFA score, renal alterations were determined only in four animals. However, it was histologically proven that all 20 animals with pyometra had renal injury. Among the parameters that differed between animals with and without sepsis, UP/CR and urea were significantly higher in animals with sepsis. With these findings, it can be inferred that the parameters used by SOFA to determine renal dysfunction do not reflect the actual functional or structural condition of this system, since they only identify changes in the advanced stage of impairment^38^. UP/CR is suggested to be a better parameter for early AKI determination in critically ill patients with suspected sepsis in the SOFA, as UP/CR >0.15 allows the AKI identification in 18 out of 20 animals with pyometra with sensitivity and specificity of 90%, similar to that described by Antunes and colleagues in humans^20^.

Regarding ultrasound examination in animals with sepsis, the corticomedullary ratio is associated with cortical and medullary region thickness. The reduction of cortical thickness is associated with chronic kidney disease, while normal or increased values for cortical thickness may occur in acute kidney disease, suggesting oedema or infiltration^27,33^. In our study, we found a reduction in the corticomedullary ratio in animals with sepsis, indicating a chronicity of renal injury probably related, as previously mentioned, to a greater inflammatory tissue reaction.

In conclusion, AKI is a very prevalent condition in patients with sepsis, derived mainly from changes in renal perfusion and inflammation and reflected by clinical, haematological, urinary, and ultrasonographic variables: RLeng/AorRatio >5.93, pulsatility index >1.53, TaMin <21.5 cm/s, TaMax <45.7 cm/s, and CortPeak <69%, with regard to perfusion changes, and UP/CR > 0.15 and serum albumin <2.94 mg/dL, with regard to inflammatory alteration. These variables were indicated as accurate (>80%) indicators of sepsis-related AKI. Based on the SOFA score, only 20% (4/20) of animals were diagnosed with renal dysfunction; nonetheless, the UP/CR was one of the parameters that correlated with sepsis status and when using the cohort value of >0.15 as indicator of AKI, which would allow classifying 90% (18/20) of animals diagnosed by histopathology; due to this finding, it is suggested to review the SOFA score parameters to identify renal failure, because serum creatinine is a poorly sensitive and late predictor when compared to a UP/CR examination of similar cost and complexity.

## Methods

This prospective clinical study including all its methods followed the recommendations of the Brazilian National Council for the Control of Animal Experimentation (CONCEA) and were approved by the Ethics Committee in the Use of Animals of the São Paulo State University (Unesp), School of Agricultural and Veterinarian Sciences, Jaboticabal, São Paulo, Brazil (protocol no. 006670/17). Corresponds to a prospective clinical case-control study, developed between March 2017 and August 2018. The tutors of the animals selected for this study were consulted, informed, and clarified regarding all details of the experiment and stated their agreement with the evaluations proposed in terms of free and informed consent. The selected patients were monitored during the experimental period and for at least 15 days. The veterinary team remained at the disposal for any intercurrence from the procedures.

Thirty-two bitches were included: 20 with pyometra (3 to 15 years old), diagnosed clinically and ultrasonographically in the Obstetric and Animal Reproduction sector of the “Governador Laudo Natel” Veterinary Hospital of FCAV-UNESP, Jaboticabal, SP, Brazil, and 12 healthy dogs (4 to 10 years old), who participated by the voluntary authorization of their tutors. The sample size was calculated (G*POWER, Universität Kiel, Germany) based on a previous study in dogs evaluating the diagnostic accuracy of the CEUS in AKI related to ischaemia^16^. This analysis indicated that an affected group of at least 15 animals and a healthy group of 10 could identify differences of at least 1.0 s in the CEUS vascular indices, a difference greater than the minimum detected in the study used as the base (1.6 s, n = 5), with significance of 5% (α = 0.05) and statistical power of 90% (1-β = 0.90).

Before the ultrasound exam, a physical examination was carried out: rectal temperature (°C) with mercury thermometer (1 minute), heart rate (HR) and respiratory rate (*f*_***R***_) by thoracic auscultation, level of consciousness by Glasgow Coma Scale (GCS) adapted for canine species^39^, and systolic arterial blood pressure (SAP) obtained in triplicate with vascular Doppler (DV610V, Medmega, São Paulo, Brazil), positioned over the metacarpal artery and vasoconstrictor cuff of appropriate size positioned on the patient’s thoracic limb.

Blood samples were also collected for blood cellular counts (erythrocytes, haemoglobin, haematocrit, mean corpuscular volume [MCV], mean corpuscular haemoglobin [MCH], mean corpuscular haemoglobin concentration [MCHC], platelets, leukocytes, basophils, eosinophils, band cells, neutrophils, lymphocytes, and monocytes), performed by impedance in an automatic haematology analyser (ABX-MICROS-ESV60, Horiba Medical, São Paulo, Brazil), and biochemical analyses (creatinine [mg/dL], urea [mg/dL], total protein [g/dL], albumin [g/dL], globulins [g/dL], alanine aminotransferase [ALT, U/L], alkaline phosphatase (ALP [U/L], total bilirubin [TBilirubin, mg/dL], conjugated bilirubin [CBilirubin mg/dL], and unconjugated bilirubin [UBilirubin, mg/dL] serum concentration), performed in a semiautomatic spectrophotometer (LABTEST, LabQuest, São Paulo, Brazil) using commercial kits of this equipment, according to the manufacturer’s guidelines.

Glycaemia (mg/dL) was measured on a portable glycosometer (G-TECH FREE, SD Biosensor, Gyeonggi, South Korea) with the remaining blood in the catheter, immediately after fixation of access in the cephalic vein. Urine samples were collected by ultrasound-guided cystocentesis for urinalysis (URIQUEST PLUS, Labtest, São Paulo, Brazil) and urinary protein/creatinine ratio (UP/CR), and arterial blood was collected from the femoral artery with heparinized syringe to measure the partial pressure of oxygen and carbon dioxide (PaO_2_ and PaCO_2_ mmHg), pH, and arterial bicarbonate concentration (CaHCO_3_ mEq/L), using an automatic blood gas analyser (OMNI-C, Roche Diagnostics GmbH, Mannheim, Germany).

Initially, these tests were used to confirm the health status of the control group, identify sepsis based on the SOFA score adapted for canine species^40^, decide appropriate therapy by the veterinary clinical team, and perform the statistical analyses.

After clinical examination, blood and urine collection, a wide abdominal trichotomy, and left cephalic vein catheterization were performed. The animals were conducted for ultrasound evaluation (ACUSON S2000, Siemens, Munich, Germany, equipped with a 4.0–9.0-MHz linear or convex transducer). Abdominal ultrasound exam was started, prioritizing to locate and carry out the evaluations described below in the uterus and both kidneys (right and left) of each patient, in longitudinal and transverse sections.

In B-mode ultrasonography, renal dimensions (length, width, and height), corticomedullary ratio (Cort/MedRatio), renal length/aorta diameter ratio (RLeng/AorRatio), and renal volume (length × height × width × 0.523) were evaluated^41–44^. Additionally, the mean cross-sectional area of the left renal artery was obtained after measuring it in triplicate during diastole and systole, and from the uterine structure, the cross-sectional uterine diameter, uterine wall thickening, and lumen diameter (cm) were evaluated.

Doppler analysis was performed after locating the renal artery using colour Doppler, and then the pulsed Doppler mode was activated ensuring that the angle between the Doppler beam and the long axis of the vessel was less than 60°. The colour gain has been adjusted to reduce excessive noise when blood flow was too slow. The calliper of the sampling window was set between 2 and 3 mm equivalent to 2/3 of the vessel diameter and positioned in the central area of the vessel. Subsequently, the spectral flow path was evaluated and recorded until quality waves were obtained with as few artefacts as possible. The following parameters were automatically obtained: systolic velocity (SystVel), diastolic velocity (DiastVel), time-averaged minimum velocity (TaMin), time-averaged maximum velocity (TaMax), pulsatility index, and resistivity index. Renal blood flow (RBF = (TaMax + TaMin/2) × renal artery area / renal volume) was calculated using the Doppler and B-mode parameter measurements, as described by Grunert and colleagues and Miyamoto and colleagues^41,45^.

The CEUS evaluation was performed in the left kidney (due to the ease of evaluation) using contrast-specific software (CADENCE [contrast pulse sequencing technology], Siemens, Munich, Germany), with a harmonic image secondary to the inverse pulse technique. Immediately after centralizing the image of the longest axis of the kidney in longitudinal section on the screen, the transducer was held steadily in the position and activated Cadence. Acoustic power, gain, depth, dynamic range, frequency, and focus were optimised before the evaluation, aiming to guarantee excellent image quality, and kept constant throughout the experiment.

An intravenous bolus of contrast medium sulphur hexafluoride (SONOVUE, Bracco, São Paulo, Brazil) 0.01 mL/kg was administered. The time of application was considered as T0, initiating the video recording for a least 120 s as described and validated for dogs^14,16,46^.

**Figure 4:**
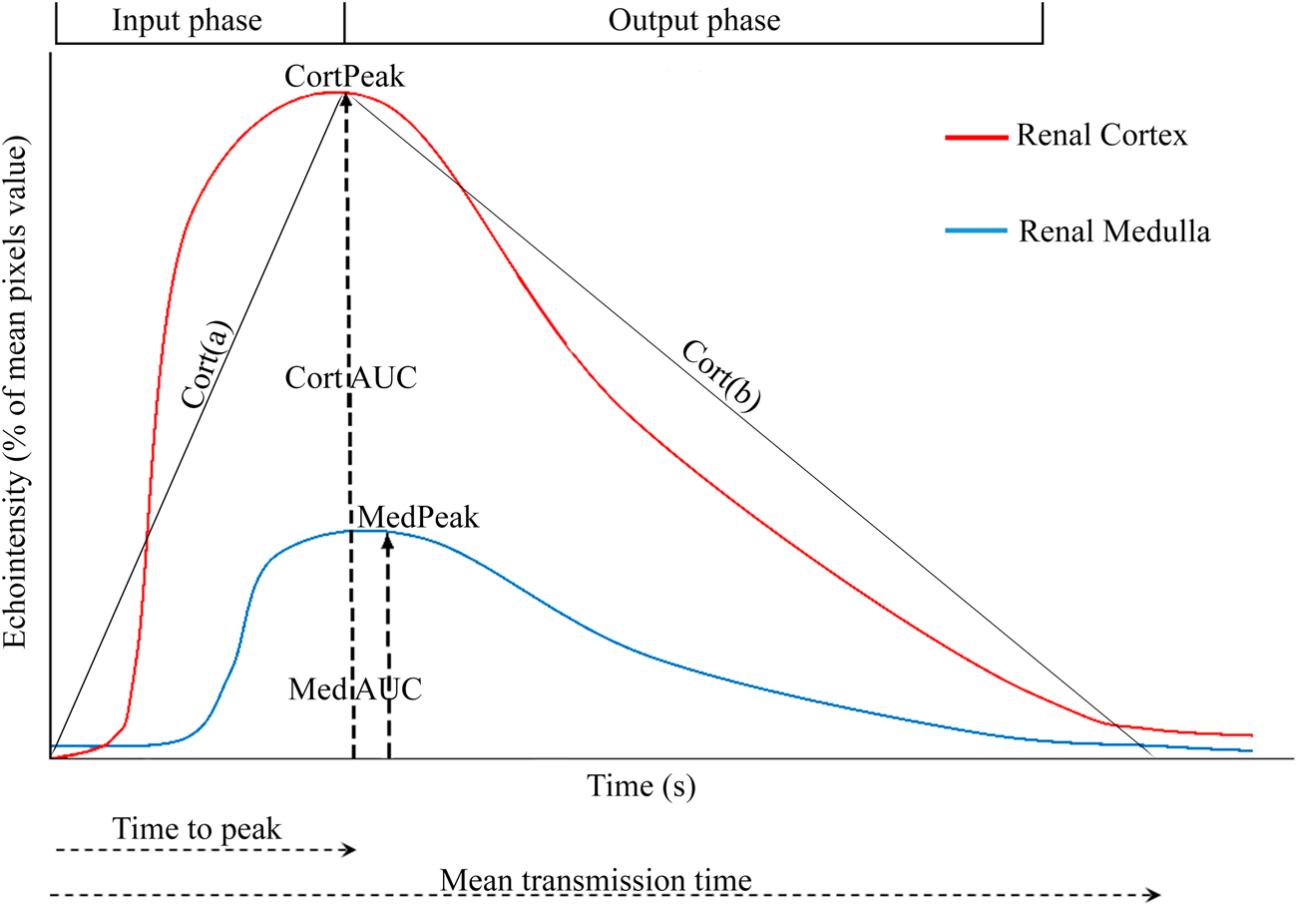
Illustration of the function that represents the time-intensity curve of the renal ultrasound contrast study after the application of ultrasonographic contrast (time 0). Parameters: peak contrast enhancement (CortPeak and MedPeak in %), time for cortical peak (CortTP in s), mean transmission time (MtT in s), area under the curve (CortAUC and MedAUC), slope of cortical input curve (Cort(a) in %/s), and the output curve (Cort(b) in %/s).

After the examination, the images were transferred to an offline analysis module (DICOM [Digital Imaging and Communications in Medicine], Rosslyn, VA, USA), in which two trained and blind evaluators for the animal clinical state defined areas of interest, initially including the largest portion of the renal cortex and then the medullary. In these areas, five subregions of interest (ROI) of approximately 1 mm² each located within the cortical or medullary parenchyma were defined as described by Wei and colleagues^13^. At this time, the processing software compares contrast time-intensity curves and automatically calculates cortical (Cort) and medullary (Med) perfusion parameters: peak contrast enhancement (CortPeak and MedPeak in % of mean pixel value), time to peak (CortTP and MedTP in s), mean transmission time (CortMtT and MedMtT in s), area under the curve (CortAUC and MedAUC), slope of the input curve (Cort(a) and Med(a) in %/s), and output curve (Cort(b) and Med(b) in %/s) (Fig. 4).

After ultrasound examination, the patients returned to the hospital routine for medical/surgical treatment, according to the institutional protocols. Once the veterinary team had finished ovariohysterectomy, profuse lavage of the abdominal cavity was performed with heated saline solution, and a biopsy of the right renal cortex was then collected with a semiautomatic biopsy needle (TRU-CUT, Velox, São Paulo, Brazil). The right kidney was chosen to perform the biopsy in order to avoid possible perfusion changes in the ultrasonographic reassessment of the left kidney, performed on the return of the animal 7 days after surgical procedure.

The tissue collected was sent in 10% formalin for histopathological analysis, performed by a veterinary pathologist experienced and blind to the animal’s conditions, in order to evaluate the presence of AKI according to the criteria of the International Veterinary Renal Pathology Initiative^47^. With the result of this exam, patients who presented tubular degeneration and necrosis were classified as positive for AKI, and the degree of lesions identified at histopathology was classified from 0 to 3, where 0 represents absence of lesion, 1 mild lesion, 2 moderate lesion, and 3 severe lesion^48^.

Statistical analysis was performed with the help of software R (R, Foundation for Statistical Computing, Vienna, Austria). The variables were compared between AKI and non-AKI patients, diagnosed using histopathological examination as a gold standard by Student’s t-test. Subsequently, the parameters that presented significant differences (p ≤ 0.01) were subjected to least squares regression analysis aiming to assess the relationship between AKI and the predictors studied, their regression coefficients, and probability ratios. At the same time, discriminative power analysis (AKI or non-AKI) was performed through receiver operating characteristic curves (ROC) and calculated the cutoff value (CV), sensitivity, specificity, likelihood ratio, accuracy, and area under curve (DiagAUC), using the logistic regression model. On these parameters, a principal component analysis (PCA) was applied, trying to identify the physiological alteration components that may explain the variability of the parameters among AKI or non-AKI patients. Additionally, correlation analysis was performed between studied variables and renal lesion degrees found on histopathological examination, by Spearman’s test. Finally, the variables evaluated were compared between sepsis-positive and sepsis-negative patients classified by the SOFA score, using Mann-Whitney U test. Except for Student’s t-test, the significance was set as p < 0.05.

## Data Availability

The datasets generated during and/or analysed during the current study are available from the corresponding author on reasonable request.
